# From Clinic to Community: An Interpretable Artificial Intelligence Framework for Enamel Caries Detection to Support Public Health Dentistry

**DOI:** 10.1055/s-0046-1816061

**Published:** 2026-02-20

**Authors:** Heba Ashi

**Affiliations:** 1 Department of Dental Public Health, Faculty of Dentistry, King Abdulaziz University, Jeddah, Saudi Arabia

**Keywords:** enamel caries classification, attention-guided feature fusion, Modified EfficientNetB0, Modified MobileNetV2, grad-CAM, dental public health

## Abstract

**Objectives:**

Dental enamel caries is among the most prevalent oral diseases worldwide. Early detection is essential, as incipient lesions can be managed with noninvasive therapies. Conventional methods, such as visual-tactile inspection and radiography, remain limited by examiner variability and reduced sensitivity for early lesions. This study aimed to develop an efficient and interpretable deep learning framework for automated classification of enamel caries at multiple severity levels, while ensuring clinical applicability and transparency.

**Materials and Methods:**

A dataset of 2,000 clinical dental images categorized as advanced enamel caries, early-stage enamel caries, and no enamel caries was curated and expanded to 12,000 images using preprocessing and augmentation. Two transfer learning models, Modified EfficientNetB0 and Modified MobileNetV2, were trained individually, then combined using an attention-guided fusion mechanism. Gradient-weighted Class Activation Mapping (Grad-CAM) was applied to provide visual interpretability.

**Statistical Analysis:**

Performance was evaluated using accuracy, precision, sensitivity, specificity, F1 score, and ROC AUC. Comparative analysis was performed across models and classifiers, with inference time assessed for clinical feasibility.

**Results:**

The Modified EfficientNetB0 and MobileNetV2 models achieved accuracies of 96.33 and 96.25%, respectively. The fused model with Random Forest demonstrated superior performance, achieving 96.92% accuracy, F1 score of 96.92, and an ROC AUC of 99.34. Misclassifications were limited to adjacent disease stages, with no severe diagnostic errors.

**Conclusion:**

The proposed framework provides accurate, interpretable, and efficient enamel caries detection. Its low inference time supports real-time clinical use, enhancing diagnostic confidence and enabling early, minimally invasive interventions. Future research should focus on multicenter validation and multimodal datasets to improve generalizability.

## Introduction

Dental caries is one of the most widespread oral health problems worldwide. At the earliest stage of dental enamel caries, damage is restricted to the enamel surface. This stage is critical; at this point, the process can be reversed through preventive strategies such as dietary modification. 1 Early intervention prevents the need for restorative procedures, reduces treatment costs, and preserves natural tooth structure. The initial clinical sign of enamel caries is a small white spot that indicates the demineralization of the enamel. 2 Without early management, the lesion progresses through the enamel into the dentin, leading to pain, pulp involvement, and possible tooth loss. 2 Therefore, accurate detection of enamel caries at an early stage is essential for prevention and minimally invasive dentistry. Traditional diagnostic methods, such as visual and tactile checkups, are often guided by the International Caries Detection and Assessment System (ICDAS), as well as radiographic techniques such as bitewing or panoramic imaging. 3 Although widely used, these approaches have limitations. Early enamel lesions are often radiographically invisible, and visual inspection highly depends on the clinician's expertise. 4 5 Dental caries persists as a major global public health crisis, exerting heavy social and financial burdens despite its preventability. 6 7 While the caries process was once viewed simply as cavitation, it is now recognized as a dynamic continuum of demineralization and remineralization, influenced by ecological shifts in biofilm composition. 8 Recently, artificial intelligence (AI) has appeared as a capable tool for dental diagnosis. Deep learning models can analyze radiographs and clinical images to identify subtle features that may be invisible to the human eye. 9 10 11 12 Generative adversarial networks (GANs) have also been employed to synthesize realistic dental images, thereby augmenting limited datasets and improving the training of deep learning models. 13 Emerging AI tools, particularly those based on deep learning, offer transformative potential: image-based detection of enamel lesions, personalized caries risk prediction, and even virtual training to enhance clinical decision-making. 7 14 15 Kühnisch et al 16 applied a CNN model to 2,417 standardized single-tooth photographs and reported 92.5% accuracy, a sensitivity of 89.6%, specificity of 94.3%, and an AUC of 96. However, inference speed was only qualitatively discussed, with no quantitative reporting of per-image runtime. Zhang et al 17 employed a single-shot multi-box detector (SSD)-based ConvNet model trained on 3,932 oral images and achieved an AUC of 85.65, with image-level sensitivity of 81.90% but a substantial drop in localization sensitivity to 64.6%, highlighting the difficulty of precise lesion detection compared with general classification. Generalizability concerns were raised by Frenkel et al. 18 They externally validated an AI model on 718 internet-sourced images, achieved 92.0% detection accuracy and a 0.702 to 0.909 classification AUC, reflecting reduced performance in real-world heterogeneous images. Beyond photographs, deep learning has also been applied to radiographs, though most studies focus on more advanced regions rather than early enamel changes. Li et al 19 utilized a modified deep learning model on 4,129 periapical radiographs, achieving an F1 score of 82.9 for caries and 82.8 for periapical periodontitis, though the model requires further validation. Estai et al 20 applied Faster R-CNN and Inception-ResNet-v2 on 2,468 bitewing radiographs, achieving an F1 score of 87 and 89 of recall and need for further validation. Tan et al 21 used CNNs on quantitative light-induced fluorescence (QLF) 9,478 images obtained via handheld devices, achieved an 88% AUC but noted a limited sensitivity of 64% for caries staging. Chaves et al 22 employed Mask R-CNN with a backbone of Swin transformer trained on 425 bitewings, achieving an F1 score of 71.9 for secondary caries, though the model requires further clinical validation. The explainable AI addresses the “black box” limitation of deep learning systems. Oztekin et al 23 applied Grad-CAM visualizations to panoramic radiographs, showing that heatmaps highlight decision-relevant regions to enhance clinical trust and utilize EfficientNetB0, DenseNet121, and ResNet50 deep learning models trained on 562 panoramic images. ResNet50 model achieved 92.00% label-wise accuracy and a 91.61% F1 score, accuracy limited to further internal validation. A summary of the literature work is shown in Table 1 .

**Table 1:** Summary of literature on AI-based enamel caries detection, highlighting datasets, methodologies, performance, and limitations

Author	Dataset/Modality	Methodology	Accuracy/Performance	Limitations
Kühnisch et al 11	2,417 single-tooth photographs	CNN	92.5%	No quantitative reporting of inference time or hardware efficiency
Zhang et al 12	3,932 oral photographs	SSD-based ConvNet	AUC: 85.65% Sensitivity: 81.90%	Localization sensitivity dropped to 64.6%; false-positive predictions
Frenkel et al 13	718 internet-sourced images	External validation of an AI model	92.0%	Reduced performance on heterogeneous images; partially correct segmentation (44.1%)
Li et al 14	4,129 periapical radiographs	Modified deep learning model	F1 score: 82.9%	Requires further validation on diverse clinical data
Estai et al 15	2,468 bitewing radiographs	Faster R-CNN and Inception-ResNet-v2	F1 score: 87%	Model requires further clinical validation
Tan et al 16	9,478 QLF images (handheld device)	CNN	AUC: 88% Validation sensitivity: 64%	Limited sensitivity for early caries staging
Chaves et al 17	425 bitewing radiographs	Mask R-CNN (Swin Transformer)	Secondary caries F1 score: 71.9%	Requires further clinical validation for primary caries
Öztekin et al 10	562 panoramic radiographs	ResNet-50 (with Grad-CAM)	Accuracy: 92.00%	Model requires further external validation

To address these limitations, this study introduces a lightweight attention-guided fusion mechanism that combines the complementary strengths of the Modified EfficientNetB0 and MobileNetV2 feature representations. Although both models are well-established architectures, our contribution lies in the way their features are combined: a simple attention-based weighting approach that selectively highlights the most informative features from each network without increasing the overall model size. This results in a more discriminative and efficient representation compared with using either model alone. In addition, Grad-CAM is incorporated to provide clear visual explanations, improving the interpretability and clinical trust of the system. By evaluating the model using multiple performance metrics and inference time, the proposed framework aims to deliver a precise, efficient, and clinically practical tool for early enamel caries classification.

## Materials and Methods

This study developed an automated diagnostic framework for the classification of dental enamel caries through a multistage computational pipeline, as illustrated in Fig. 1 . The workflow commenced with the curation of a clinical image dataset comprising 2,000 low-resolution dental images, categorized into advanced enamel caries, early-stage enamel caries, and no enamel caries. To enhance feature visibility and ensure efficiency, the dataset underwent a rigorous preprocessing stage employing histogram equalization, CLAHE, and adaptive thresholding, followed by an augmentation protocol using the Albumentations Library to increase data diversity and prevent overfitting. Two deep learning architectures, Modified EfficientNet-B0 and MobileNetV2, were developed via transfer learning. Each model featured a custom classification head with a 512-unit dense layer for deep feature extraction and was trained in a two-stage process involving initial frozen backbone training and subsequent fine-tuning. The discriminative features extracted from both models were then synergistically integrated using an attention-guided fusion mechanism, which adaptively weighed the most salient features from each network to form a unified, highly discriminative representation without increasing dimensionality. The final fused features were classified using a suite of machine learning classifiers. For model evaluation, only the original test images were used without any augmentation to ensure unbiased performance assessment. To ensure clinical transparency and interpretability, the decision-making process of the models was elucidated using gradient-weighted class activation mapping (Grad-CAM), which generated visual heatmaps highlighting the specific image regions most influential for the classification of each caries label. The selection of reporting checklists was informed by a recent integrative review consolidating major AI reporting frameworks, including CONSORT-AI, TRIPOD-AI, PRISMA-AI, CLAIM, STARD-AI, and DECIDE-AI, with specific relevance to dental research. 24 Based on this guidance, the present study adheres to the Checklist for Artificial Intelligence in Medical Imaging (CLAIM) 2024 25 and the STARD 2015 guidelines 26 to ensure methodological rigor, transparency, and reproducibility.

**Fig. 1:**
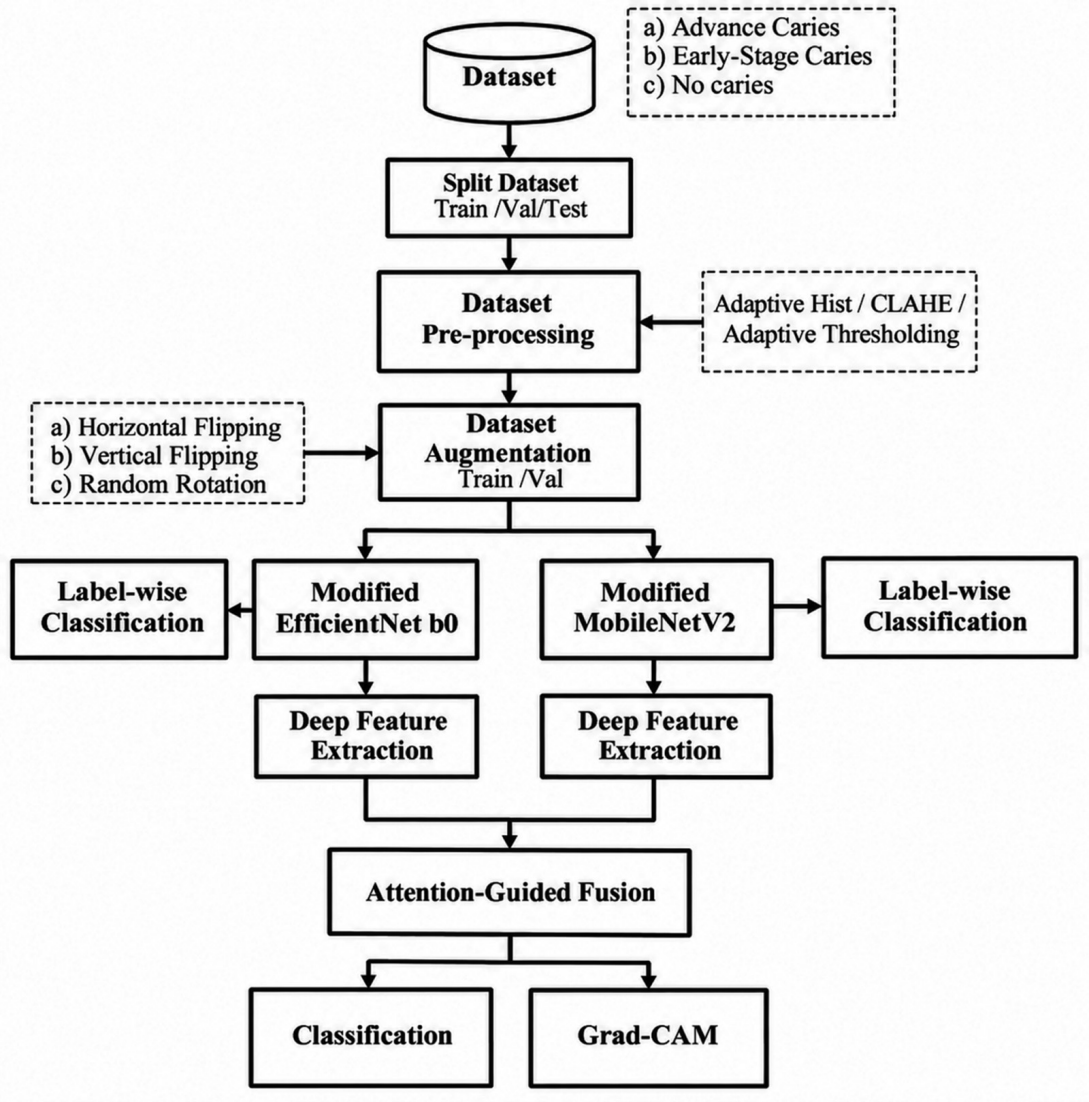
Proposed pipeline for enamel caries detection, integrating preprocessing, augmentation, dual deep feature extraction from EfficientNet-B0 and MobileNetV2, attention-guided fusion, classification, and Grad-CAM explainability.

### Dataset Collection

In this experiment, a publicly available dataset of enamel caries is utilized. The images were acquired from several medical clinics located in Rajshahi and Dhaka, Bangladesh. 27 These images represent a heterogeneous population of patients to ensure the variability in dental characteristics and enhance the generalizability of the dataset. The dataset is categorized into three categories: advanced enamel caries, early-stage enamel caries, and no enamel caries. A total of 2,000 original images in JPG format are described in Table 2 . It is a valuable resource for the development and evaluation of an automated diagnostic framework in dental image analysis, and it does not require ethical approval since it is publicly available for research purposes. 27 The dataset was divided into two main partitions: a training set, a validation set, and a test set. A total of 1,200 original images were reserved as the test set, and the remaining 800 images were used for training and internal validation. From the 800 training images, 20% were used as the validation set to monitor model performance during training.

**Table 2:** Distribution of images in the dental caries dataset

Labels	Number of images (jpg)
Advanced enamel caries	800
Early-stage enamel caries	800
No enamel caries	400
**Total**	**2000**

### Inclusion and Exclusion Criteria

To ensure consistency, only images that met clear clinical and visual standards were included.

#### Inclusion Criteria

- Intraoral photographs showing teeth and enamel surfaces clearly.
- Images belong to one of the three predefined categories.
- JPG images of 224 × 224 × 3 resolution.
- Fully anonymized images without any identifiable information.

#### Exclusion Criteria

- Blurred, dark, overexposed, or low-quality images.
- Images containing restorations, orthodontic wires, or artifacts covering enamel surfaces.
- Damaged or incomplete image files.
- Duplicate images or images appearing across multiple data subsets.

### Dataset Preprocessing Techniques

In this study, image preprocessing techniques were applied to enhance the visibility of dental enamel caries and to reduce variability caused by illumination differences. Three enhancement methods were employed in this study. First, histogram equalization was utilized to improve global contrast, to redistribute pixel intensities, and to enhance the visibility of enamel defects. 28 Second, CLAHE was applied to provide localized contrast enhancement while preventing noise over-amplification, which is advantageous in medical images. 29 Finally, adaptive thresholding was employed to segment structural regions to compute the pixel-level thresholds adaptively over localized areas, ensuring reliability against nonuniform illumination. 30 The effects of images after the preprocessing techniques applied are illustrated in Fig. 2 .

**Fig. 2:**
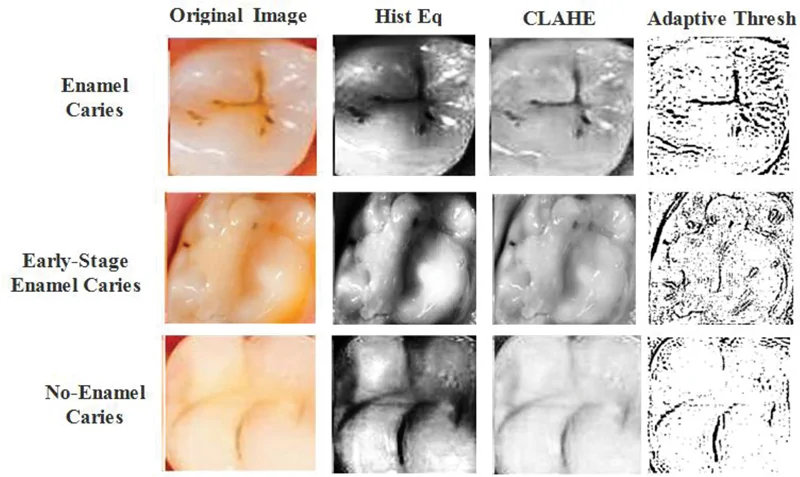
The preprocessing stage demonstrates contrast enhancement using histogram equalization, localized feature improvement with CLAHE, and structural boundary extraction through adaptive thresholding. These preprocessing techniques collectively contribute to improved feature representation for model training.

### Dataset Augmentation

The augmentation pipeline was implemented using the Albumentations Library, 31 which provides efficient and diverse image transformations widely used in medical image analysis. The applied transformations included geometric operations (horizontal and vertical flipping, random rotation), intensity-based adjustments brightness and contrast modification, gamma correction, histogram equalization, and CLAHE. Additionally, morphological operations and adaptive thresholding were employed to enhance structural features, 32 and random cropping was performed, followed by resizing to a fixed resolution of 224 × 224 pixels to maintain uniformity. The summary of the augmentation process is presented in Table 3 .

**Table 3:** Dataset distribution for dental caries detection

Labels	Original no. Of images	After augmentation	Augmented train set	Augmented validation set	Original test set
Early-stage enamel caries	800	4,000	3,200	400	500
No enamel caries	400	4,000	3,200	400	200
Advanced enamel caries	800	4,000	3,200	400	500
**Total no. of images**	**2,000**	**12,000**	**9,600**	**1,200**	**1,200**

**Note:** Test set was separated from the original dataset without augmentation, while the training and validation sets were created using augmentation.

### Modified EfficientNet-B0

For the classification of dental enamel caries, transfer learning-based strategy was developed by using EfficientNetB0 as the feature extractor. The pretrained EfficientNetB0 network was adopted as the base architecture with pretrain weights, with its convolutional backbone frozen during the initial training stage. A custom classification head was then appended to adapt the model for the specific multi-class dental caries classification task. The architecture consisted of the following components: the EfficientNetB0 convolutional base, excluding its original fully connected layers, a global average pooling layer to reduce feature dimensionality, 33 a dropout regularization, and a custom dense feature extraction layer with 512 units, ReLU activation function, and then a batch normalization layer was applied to improve generalization and stabilize training. 34 Finally, a fully connected SoftMax layer was used for multiclass probability prediction. 35 Table 4 summarizes the architecture of the proposed model along with its associated hyperparameters.

**Table 4:** Proposed model architecture and training hyperparameters

Layer/Component	Configuration/Hyperparameter
Input layer	Images size 224 × 224 × 3
Base model	EfficientNetB0 (pretrained weights with no top layers)
Global average pooling	2D GAP for feature maps
Dropout (regularization)	0.30%
Dense (feature extraction layer)	512 units, linear activation before BN/ReLU
Batch normalization	Applied after the feature extraction layer
Activation	ReLU
Dropout (regularization)	0.40%
Dense (classification Layer)	Softmax activation, number of units = 3
Optimizer—Stage 1	Adam, learning rate = 10 ^−3^ (frozen base)
Epochs—Stage 1	20
Optimizer—Stage 2	Adam, learning rate = 10 ^−4^ to fine-tuned model
Epochs—Stage 2	80
Fine-tuned layers	Last 60 layers of EfficientNetB0 unfrozen
Batch size	32
Data augmentation	Rotation (25°), width/height shift (0.15), zoom (0.20), horizontal flip
Early stopping	Patience 8 to 10 depending on the training stage
ReduceLROnPlateau	Factor 0.20, patience 4 to 5, min LR = 10 ^−7^

During Stage 1, freezing, the base model layers and custom classification head are trained to learn high-level discriminative features. In Stage 2, the last 60 layers of EfficientNetB0 were unfrozen for fine-tuning while maintaining a lower learning rate for domain-specific feature adaptation. 36 The training process was monitored using accuracy and loss curves, which are presented in Fig. 3 . These curves demonstrate the convergence behavior of the model and the effectiveness of the staged training procedure in reducing overfitting.

**Fig. 3:**
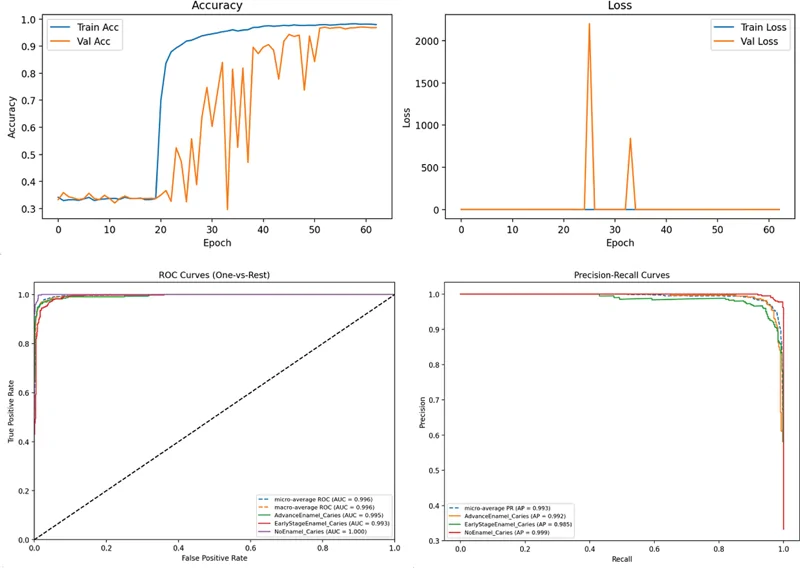
Performance of the proposed EfficientNetB0-based model for enamel caries classification. ( **A** ) Training and validation accuracy across epochs. ( **B** ) Training and validation loss trends. ( **C** ) Receiver operating characteristic (ROC) curves with micro- and macro-averaged AUC values. ( **D** ) Precision, recall (PR) curves showing high average precision across all classes.

### Modified MobileNetV2 Architecture

A second deep learning model was developed using MobileNetV2 as the feature extractor. Similar to the EfficientNetB0-based pipeline, applied a transfer learning strategy with a two-stage training process, as shown in Table 5 . The pretrained MobileNetV2 weights were used as the convolutional backbone, with a custom classification head designed to enhance discriminative feature learning for enamel caries detection. The model consisted of the following major components: the MobileNetV2 base, excluding its top fully connected layers, a global average pooling layer to aggregate features, dropout layers for regularization, 37 a custom dense feature extraction layer with 512 units, batch normalization, ReLU activation, and a final SoftMax layer to use for multi-class classification. 38 Compared with the standard MobileNetV2 classifier head, the following modifications were introduced:

**Table 5:** Architecture and hyperparameters of the proposed Modified MobileNetV2 model

Layer/Component	Configuration/Hyperparameter
Input layer	Images size = 224 × 224 × 3
Base model	MobileNetV2 pretrained weight without top layers
Global average-pooling layer	2D GAP applied to feature maps
Dropout (regularization)	30 percent
Dense (feature extraction layer)	512 units, linear activation before BN/ReLU
Batch normalization	Applied after feature extraction layer
Activation	ReLU
Dropout (regularization)	40 percent to prevent overfitting
Dense (classification layer)	SoftMax activation, number of units = 3
Stage 1: Optimizer	Adam, learning rate = 10 ^−3^ (frozen base)
Stage 1: Epochs	20
Stage 2: Optimizer	Adam, learning rate = 10 ^−4^ (fine-tuned)
Stage 2: Epochs	80
Fine-tuned layers	Last 60 layers of MobileNetV2 unfrozen
Batch size	32
Data augmentation	Rotation (25°), width and height shift, zooming and horizontal flip
Early stopping	Patience 8 to 10 depends on model training stage
ReduceLROnPlateau	Factor 0.2, patience 4 to 5, min LR = 10 ^−7^

- Inserted a custom dense feature extraction layer (512 units) before the classification to improve feature representation.
- Applied double dropout regularization of 0.3 and 0.4 at different stages to reduce overfitting. 39
- Added batch normalization with ReLU block after the feature extraction layer to stabilize training. 40
- Frozen backbone training, Stage 1, and then fine-tuning of the last 60 layers, Stage 2.
- Applied advanced data augmentation, such as rotation, shift, zoom, and flipping, to improve model generalization. 41

The model's training and validation performance are shown in Fig. 4 .

**Fig. 4:**
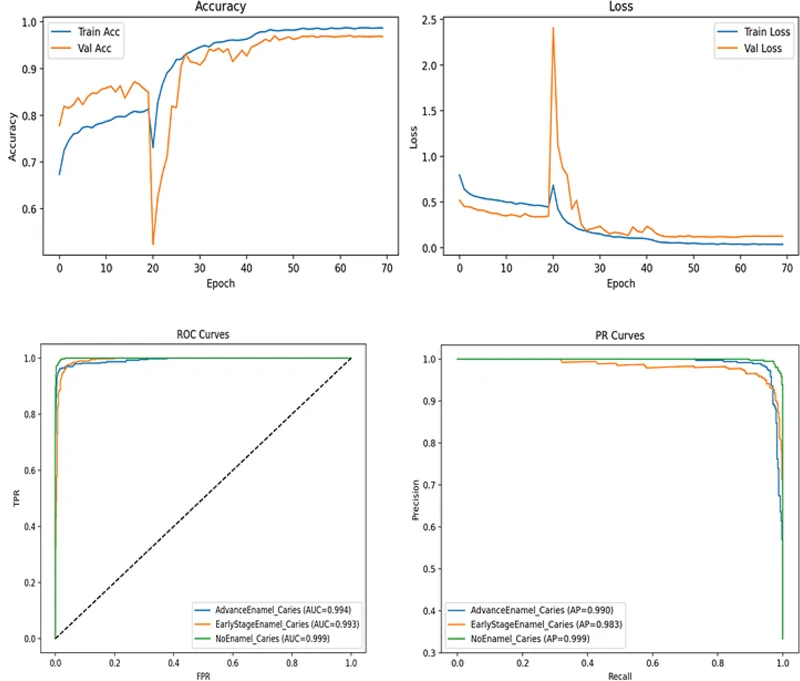
Accuracy curves demonstrate the staged improvement during transfer learning. ( **B** ) Loss curves indicate convergence with reduced overfitting. ( **C** ) ROC curves illustrate high area under the curve (AUC) values across all classes. ( **D** ) Precision and recall (PR) curves confirm high average precision (AP) scores for enamel caries detection.

### Feature Extraction

Deep feature extraction was conducted using both MobileNetV2 and the Modified EfficientNet-B0 models by isolating the 512-dimensional dense layer before classification. For MobileNetV2, the feature train set shape is 9,600 × 512, the validation set is 160 × 512, and the test set is 1,200 × 512. Similarly, for EfficientNetB0, feature dimensions are 9,600 × 512 for training, 160 × 512 for the validation set, and 1,200 × 512 for the test sets. All features were generated with shuffle set to *False* to maintain alignment between features and labels.

### Attention-Guided Feature Fusion

To integrate the features extracted from the Modified MobileNetV2 and EfficientNetB0 models, we applied a simple attention-guided fusion mechanism. In this approach, each feature vector is given a small learnable weight that indicates how useful that feature set is for the final prediction. The mechanism assigns slightly higher weight to the model that provides more relevant information for a particular image, allowing the combined feature representation to benefit from the strengths of both networks.

Unlike simple concatenation or averaging, this fusion strategy selectively highlights the more informative features while reducing overlapping or redundant information. Importantly, the final fused feature size remains the same 512 dimensions, so no extra computational cost is added. This lightweight attention-based fusion helps create a more balanced and discriminative representation, which improves the overall reliability of the enamel caries classification.

### Explainable AI with Grad-CAM

In this study, Grad-CAM was applied to images from three categories: advanced, early, and no caries. For each image, a heatmap was superimposed onto the original radiograph, producing an intuitive visualization of the model's focus. For example, in cases of early-stage enamel caries, the Grad-CAM visualization highlighted localized regions of enamel discoloration, whereas in advanced caries, larger lesion areas were emphasized, as shown in Fig. 5 . In the absence of caries, the model predominantly focused on intact enamel structures.

**Fig. 5:**
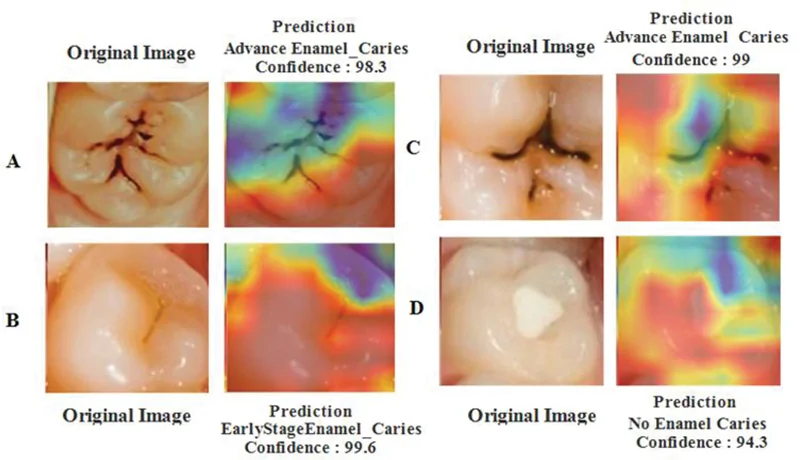
Model interpretability using Grad-CAM. Heatmaps demonstrate the model's focus on large lesions for advanced caries ( **A, B** ), a precise region for early-stage caries ( **C** ), and the absence of a focused area on healthy enamel ( **D** ), correlating with the high prediction confidence scores.

## Results

This experiment was conducted on the Kaggle cloud-based platform. The computational workload was executed on a Kaggle-provided environment equipped with a high-performance NVIDIA P100 GPU accelerator, 32 GB of RAM, and an Intel(R) Core (TM) i7–1065G7 CPU @ 1.30- to 1.50-GHz processor. This reliable hardware configuration for evaluating deep learning models significantly reduces the time required for processing large datasets and complex computations. All models and analyses were implemented using the Python programming language.

### Performance Evaluation Parameters

The quantitative evaluation of the framework performance conducted to use these metrics, such as accuracy (Acc), precision, recall, F1 score, and AUC-ROC, was derived from the constituent elements of the confusion matrix, such as true positives, false positives, true negatives, and false negatives. The formal definitions and mathematical formulations for each of these performance parameters are provided in Table 6 .

**Table 6:** Definitions and formulas for key classification performance metrics

Metric	Formula
Accuracy (Acc)	(TP + TN) / (TP + TN + FP + FN)
Precision (PPV)	TP / (TP + FP)
Recall (Sensitivity)	TP / (TP + FN)
F1 score	2 × (Precision × Recall) / (Precision + Recall)
ROC AUC	Area Under the Receiver Operating Characteristic Curve

(TP: True Positives, TN: True Negatives, FP: False Positives, FN: False Negatives)

### Class-Wise Classification

The diagnostic performance of the two modified deep learning architectures, EfficientNetB0 and MobileNetV2, was evaluated on a test set of 1,200 dental images. Both models demonstrated exceptional and highly comparable efficacy in the automated classification of enamel caries. The class-wise breakdown of precision, recall, and F1 score, along with aggregate metrics, is presented in Table 7 .

**Table 7:** Comparative diagnostic performance of Modified EfficientNetB0 and MobileNetV2 models for the classification of enamel caries

Model	Classes	Precision	Recall	F1 score	Support
**Modified EfficientNetB0**	Advanced enamel caries	96.72	95.75	96.23	400
	Early-stage enamel caries	94.51	94.75	94.63	400
	No enamel caries	97.77	98.50	98.13	400
	**Accuracy**			**96.33**	**1,200**
	**Macro average**	96.33	96.33	96.33	1,200
	**Weighted average**	96.33	96.33	96.33	1,200
**Modified MobileNetV2**	Advanced enamel caries	97.44	95.25	96.33	400
	Early-stage enamel caries	94.06	95.00	94.53	400
	No enamel caries	97.28	98.50	97.89	400
	**Accuracy**			**96.25**	**1,200**
	**Macro average**	96.26	96.25	96.25	1,200
	**Weighted average**	96.26	96.25	96.25	1,200

As shown in Table 7 , both Modified EfficientNetB0 and MobileNetV2 (96.33 and 96.25%, respectively) achieved excellent accuracy. With the highest F1 scores in the no caries class and reliable performance in early-stage lesions, both models demonstrated balanced, unbiased, and highly effective capabilities for automated enamel caries detection and classification.

As presented in Fig. 6 , both Modified MobileNetV2 and EfficientNetB0 models achieved high diagnostic accuracy. MobileNetV2 correctly classified 381 advanced, 380 early-stage, and 394 no-caries cases, while EfficientNetB0 showed slightly superior performance for advanced lesions, with comparable results across other classes, confirming reliability in enamel caries detection.

**Fig. 6:**
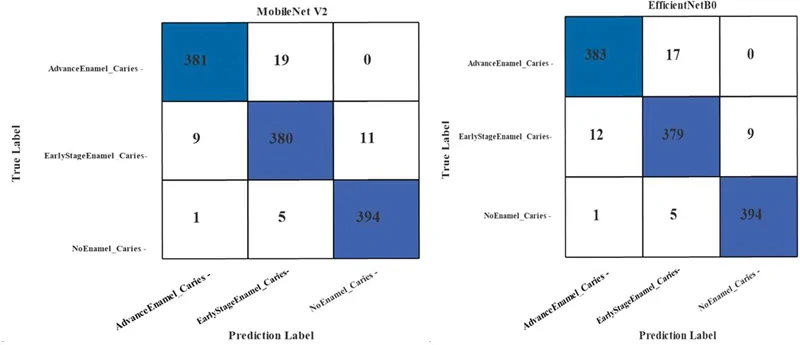
The matrix illustrates the model's classification patterns, confirming its high accuracy and specific confusion profiles between caries stages.

### Deep Feature Extraction Results

The performance of various classifiers utilizing deep features extracted from the Modified EfficientNetB0 and MobileNetV2 networks is summarized in Table 8 .

**Table 8:** Comprehensive performance evaluation of classifiers using deep features extracted from Modified EfficientNetB0 and MobileNetV2 architectures

Feature source	Classifier	Train time (s)	Prediction time (s)	Accuracy	Precision	Recall	F1 score	ROC AUC
**EfficientNetB0**	SVM cubic	4.6473	0.1301	95.83	95.85	95.83	95.81	98.75
	KNN fine	0.0037	0.2409	96.08	96.11	96.08	96.09	97.06
	**KNN Medium**	**0.0029**	**0.2127**	**96.67**	**96.68**	**96.67**	**96.67**	**98.63**
	Decision tree	2.9580	0.0006	95.67	95.66	95.67	95.66	97.40
	Fine tree	4.4815	0.0007	95.92	95.92	95.92	95.92	97.22
	Naive Bayes	0.0350	0.0159	96.33	96.34	96.33	96.33	98.35
	Random forest	11.6900	0.0144	96.25	96.26	96.25	96.25	99.33
	AdaBoost	33.9521	0.0235	95.67	95.70	95.67	95.68	99.31
	Neural Net	5.0523	0.0029	96.25	96.24	96.25	96.24	99.44
	Logistic regression	6.8525	0.0098	96.50	96.49	96.50	96.50	99.43
**MobileNetV2**	SVM cubic	3.5391	0.0616	96.25	96.28	96.25	96.25	98.98
	KNN fine	0.0030	0.2296	96.75	96.81	96.75	96.76	97.56
	KNN medium	0.0030	0.2098	96.42	96.44	96.42	96.42	98.33
	Decision tree	2.6495	0.0007	96.50	96.53	96.50	96.51	97.93
	Fine tree	3.5945	0.0008	96.17	96.20	96.17	96.17	97.24
	Naive Bayes	0.0363	0.0102	96.50	96.52	96.50	96.50	98.40
	Random forest	10.9025	0.0141	96.67	96.69	96.67	96.67	99.05
	**AdaBoost**	**34.6061**	**0.0238**	**96.75**	**96.82**	**96.75**	**96.76**	**99.10**
	Neural Net	4.1114	0.0025	96.33	96.36	96.33	96.34	99.03
	Logistic regression	14.5117	0.0021	95.92	95.95	95.92	95.92	99.34

As evidenced in Table 8 , the KNN Medium classifier with Modified EfficientNetB0 features and the KNN Fine and AdaBoost models with Modified MobileNetV2 features achieved the highest classification accuracy. All evaluated classifiers attained an F1 score greater than 95%, indicating reliable and balanced precision and recall characteristics. The ROC AUC values consistently exceeded 97%, confirming excellent model discriminative ability. The AdaBoost classifier achieved high accuracy, correctly classifying 382 advanced enamel caries, 389 early-stage cases, and 390 no-caries cases, with all errors conservatively assigned to adjacent categories. Similarly, the KNN Medium model correctly identified 384 advanced, 383 early-stage, and 393 no-caries cases, with minimal misclassifications mainly between adjacent stages. Both models demonstrated strong reliability, with no severe diagnostic errors, supporting their suitability for early enamel caries detection in clinical settings, as shown in Fig. 7 .

**Fig. 7:**
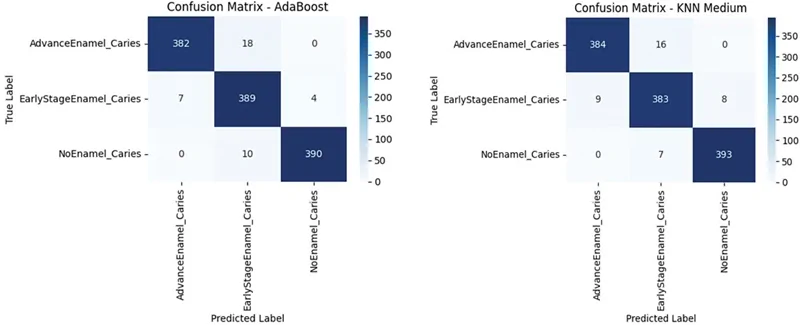
The AdaBoost and KNN Medium Classifier confusion matrix illustrates the model's classification patterns, confirming its high accuracy.

### Attention-Guided Feature Fusion Results

The implementation of an attention-guided feature fusion mechanism, integrating deep features from the Modified EfficientNetB0 and MobileNetV2 architectures, yielded a significant enhancement in diagnostic performance. The complete results of various classifiers operating on these fused features are presented in Table 9 .

**Table 9:** Performance evaluation of classifiers utilizing attention-guided fused features from EfficientNetB0 and MobileNetV2

Classifier	Train time (s)	Prediction time(s)	Accuracy	Precision	Recall	F1 score	ROC AUC
Random forest	9.7483	0.0149	96.92	96.94	96.92	96.92	99.34
**KNN medium**	**0.0044**	**0.1718**	**96.75**	**96.77**	**96.75**	**96.75**	**98.46**
Naive Bayes	0.0570	0.0085	96.67	96.67	96.67	96.67	98.68
Neural Net	6.0834	0.0035	96.17	96.22	96.17	96.17	99.19
KNN fine	0.0045	0.1629	96.50	96.52	96.50	96.50	97.37
SVM cubic	3.5067	0.0774	96.00	96.04	96.00	95.99	99.12
AdaBoost	34.3369	0.0401	94.83	95.12	94.83	94.88	99.11
Logistic regression	11.9684	0.0014	94.83	95.01	94.83	94.87	99.02
Fine tree	2.5433	0.0013	87.83	89.27	87.83	87.69	90.88
Decision tree	2.3665	0.0013	86.92	88.78	86.92	86.78	88.67

As shown in Table 9 , the attention-guided fusion approach significantly enhanced diagnostic performance, with the random forest classifier achieving an accuracy of 96.92%, an F1 score of 0.9692, and a ROC AUC of 0.9934. This improvement was obtained without added computational cost, as the fused feature vector remained at 512 dimensions. The confusion matrix in Fig. 8 further validated the model's robust classification across all enamel caries categories, confirming its reliability and clinical applicability.

**Fig. 8:**
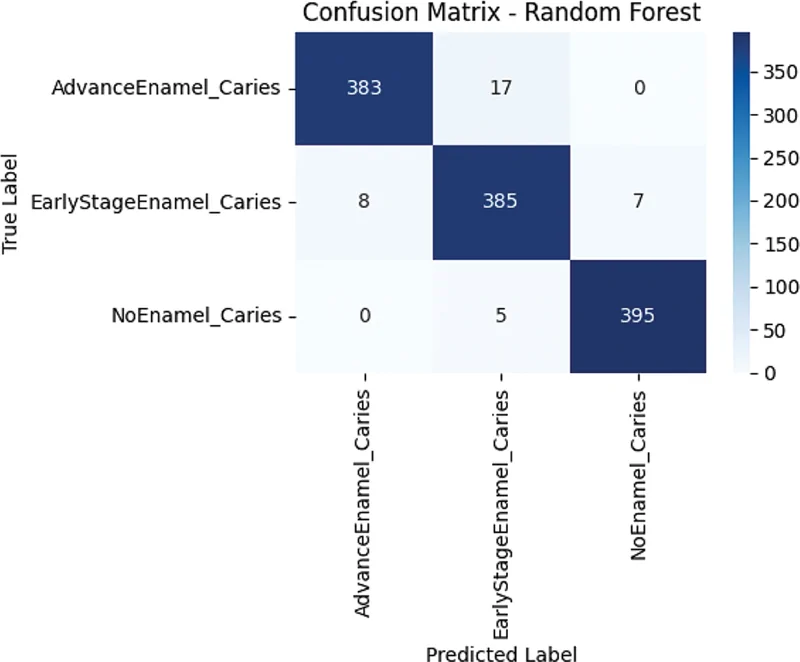
Confusion matrix of the Random Forest classifier achieved the highest accuracy.

As illustrated in Fig. 8 , the model demonstrated exceptional proficiency. For advanced enamel caries, 383 cases were correctly identified, with the remaining 17 misclassifications all conservatively predicted as the less severe early-stage caries. In classifying early-stage enamel caries, the model achieved 385 correct predictions, with errors distributed as 8 misclassifications as advanced and 7 as no caries. The performance for the no enamel caries class was near perfect, with 395 correct identifications; all five errors were predicted as the most conservative early-stage condition.

### Statistical Significance Analysis

To quantitatively validate that the performance improvement of the proposed attention-guided feature fusion model was statistically significant and not due to random chance, two rigorous statistical tests were employed: the paired Student's *t* -test and McNemar's test. These tests compare the proposed model against the best-performing baseline models, Modified EfficientNetB0 and MobileNetV2, to ascertain the significance of the observed differences in classification outcomes.

#### *T* -Test Results

A *t* -test was conducted to compare the accuracy distributions obtained from cross-validation (10-fold) of the proposed model and the baseline models. The null hypothesis (H0) stated that there was no significant difference in the mean accuracy between the models, 42 while the alternative hypothesis (H1) stated that a significant difference existed. 42 Results of the paired Student's *t* -test for model accuracy is shown in Table 10 .

**Table 10:** Results of the paired Student's *t* -test for model accuracy comparison

Comparison	*t* -Statistic	*p* -Value	Mean difference	Conclusion ( *α* = 0.05)
Proposed versus EfficientNetB0	4.32	0.0018	0.0067	Reject H0
Proposed versus MobileNetV2	3.89	0.0036	0.0058	Reject H0

As shown in Table 10 , the comparisons yielded *p* -values well below the significance level of *α* = 0.05. This provides strong evidence to reject the null hypothesis, confirming that the difference in mean accuracy between the proposed attention-guided fusion model and each baseline model is statistically significant.

#### McNemar's Test Results

McNemar's test was performed on the prediction outcomes of the proposed model and the best baseline MobileNetV2 with AdaBoost to evaluate the significance of the disagreement in their classifications. This test is particularly suited for paired nominal data and is based on a chi-squared ( *χ* ^2^ ) statistic derived from the counts of discordant pairs. The contingency ( Table 11 ) for the test is as follows.

**Table 11:** Contingency table comparing the performance of the proposed model with the baseline model

	Proposed model: Correct	Proposed model: incorrect
Baseline model: correct	1150 (a)	25 (b)
Baseline model: incorrect	45 (c)	20 (d)

The resulting *p* -value is 0.0231. A statistically significant difference in error rates between the two models, *p* < 0.05, led to the rejection of the null hypothesis of marginal homogeneity. This result suggests that the performance disparity is unlikely to have occurred by chance. The McNemar test revealed a markedly greater number of discordant pairs, 43 where the proposed model was correct and the baseline model incorrect ( *n* = 45) than the reverse ( *n* = 25). This asymmetry in misclassifications demonstrates that the proposed model's improvement is both systematic and statistically significant.

## Discussion

The present study introduced an attention-guided fusion framework that integrates features from Modified EfficientNetB0 and MobileNetV2 models to improve enamel caries detection. The framework demonstrated superior diagnostic performance, with an overall accuracy of 96.92% and an ROC AUC of 0.9934. These results are highly competitive compared with previously published work on enamel and dental caries detection, as shown in Table 12 . Kühnisch et al 16 applied MobileNetV2 on intraoral images and reported an accuracy of 92.5% with an AUC of 0.96, but their study did not provide model efficiency details. Zhang et al 17 used SSD on consumer-grade images, achieving a lower performance AUC of 0.856 with a significant reduction in localization sensitivity of 64.6%. Frenkel et al 18 validated an AI-based photographic system and achieved an accuracy of 92%, though class-wise AUC varied widely, from 0.70 to 0.91, showing inconsistency across lesion types. Similarly, Estai et al 20 and Chaves et al 22 achieved 87% acc on radiographs, but their focus was mainly on dentinal or secondary dental caries ( Fig. 9 ).

**Table 12:** Comparison of AI-based methods for caries detection, showing datasets, methodologies, and key performance metrics

Author	Dataset	Methodology	Accuracy	Precision	Recall	F1 score	AUC	Prediction time(s)
Kühnisch et al 11	2,417 intraoral photos	MobileNetV2 (CNN)	92.5%	–	89.6%	–	0.96	–
Zhang et al 12	3,932 consumer-grade photos	SSD-based ConvNet	–	–	81.90%	–	0.856	–
Frenkel et al 13	718 web images	AI-based model	92.0%	–	–	–	0.702–0.909	–
Li et al 14	4,129 periapical radiographs	Modified CNN	–	0.82	0.83	0.829	0.88	–
Estai et al 15	2,468 bitewing radiographs	Inception-ResNet-v2	0.87	0.86	0.89	0.87	–	–
Chaves et al 17	425 bitewing radiographs	Mask R-CNN (Swin-T)	–	–	–	0.689	–	–
Proposed study	Caries-Spectra 2000 low-resolution enamel caries images	**Attention-guided fusion**	**96.9%**	**96.9%**	**96.9%**	**96.9%**	**99.34%**	**0.0149**

**Fig. 9:**
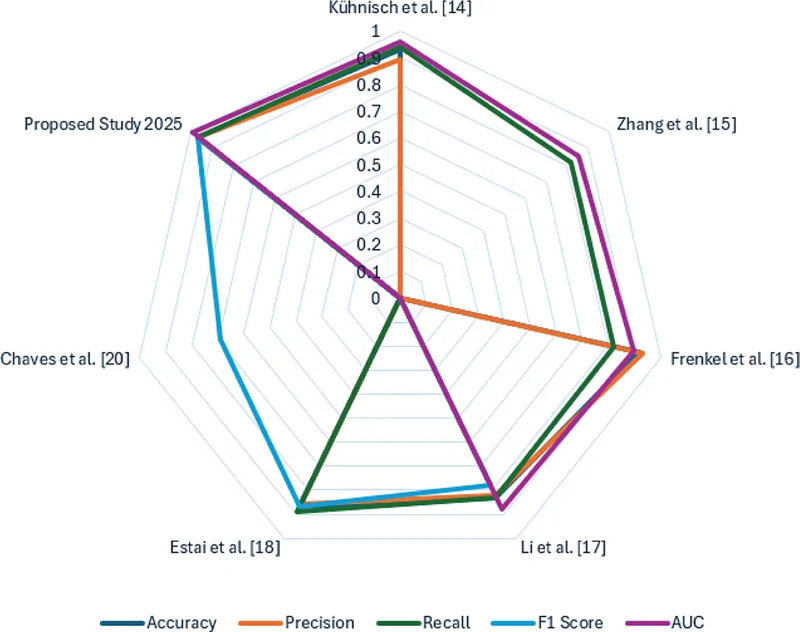
Comparative performance metrics of the proposed deep learning architecture against existing methodologies in medical image analysis. Performance evaluation includes accuracy, precision, recall, F1 score, and area under the curve (AUC) metrics across multiple benchmark studies.

Beyond these benchmarks, recent literature highlights the broader clinical and public health potential of deep learning. 44 Deep learning models have demonstrated strong accuracy in detecting dental caries from images, such as smartphone images, with promising sensitivity and specificity for cavitated lesions. 15 Furthermore, machine learning algorithms applied to survey and demographic data have reliably predicted individuals and adolescents at high risk of developing caries. This enables targeted early interventions and optimizes resource allocation in public health systems. 45 46

Policy-maker benefits:

- Enables data-driven resource distribution to predict high-risk populations and focus on preventive care where it is most needed.
- Supports evidence-based policymaking, informs the national strategies for early detection programs, and preventive dentistry.
- Advances a paradigm shift in dental practice models, moving from reactive drill and fill approaches toward proactive, risk-based, and minimally invasive care protocols.

### Clinical Significance and Implications for Practice

The clinical significance of this study lies in its ability to detect early enamel caries with high accuracy at the initial stage. By integrating explainable AI, our framework shows visual maps of the regions that influence the prediction, making the system transparent and easy to trust. The model is efficient and accurate, with low prediction time. This makes it suitable for real-time chairside use where dentists need quick support without heavy computing resources. These features increase diagnostic confidence for general practitioners, promote early preventive interventions, and extend access to caries detection in resource-limited settings.

## Conclusion

This study proposed and validated a novel attention-guided feature fusion framework for the automated classification of enamel caries. By synergistically integrating deep features from Modified EfficientNetB0 and MobileNetV2 architectures, the model achieved a superior diagnostic performance, with a peak accuracy of 96.92% and an ROC AUC of 0.9934. Statistical significance testing confirmed that this improvement over strong baseline models was not due to random chance. Embedding deep learning in dental enamel caries management has immense potential to improve diagnostic accuracy, facilitate early intervention, and reshape public health policy through predictive analytics and targeted care delivery. Supporting early detection and minimally invasive treatment strategies, such as frameworks, can help reduce the global burden of caries while providing policymakers with efficient evidence to guide preventive health initiatives.

## Limitations

Despite the promising results, this study has several limitations. The primary limitation is the constraint imposed by the current availability of public datasets. There is a notable absence of a large, publicly available, and expertly annotated dataset for enamel caries that includes the precise clinical classifications, such as advanced, early stage, and no caries, used in this work. Consequently, the proposed framework was trained and validated on a dataset of clinical images. Furthermore, the model's applicability is inherently limited to the detection of visible, surface-level enamel changes, and cannot be generalized to subsurface or proximal lesions typically diagnosed through radiographic evaluation. A significant methodological limitation is the lack of external validation on an independent, multicenter dataset, which is currently not available for the specific class definitions used in this study. This absence affects the assessment of the model's generalizability and real-world reliability.

## Future Work

Future research directions will focus on addressing these limitations and expanding the model's clinical utility. The foremost priority is to perform a rigorous external validation of the model. With the current unavailability of a publicly accessible dataset with compatible clinical classifications, a key immediate step will be to prospectively collect a new, multicenter clinical image dataset to serve as an external test set. This will allow for a thorough assessment of the model's reliability and generalizability beyond the internal validation performed in this study. Concurrently, we will pursue collaborations with dental institutions to assemble a larger and more diverse multimodal dataset, encompassing both clinical and radiographic images with expert annotations. This will enable the development of a next-generation model capable of fused multimodal analysis (clinical + radiographic), which would represent a significant advancement toward a comprehensive automated diagnostic system. Beyond technical development, future work should also emphasize the following:

- AI tools validate across diverse populations and imaging devices to ensure generalizability and equity.
- Integration with public dental health systems to support risk-based preventive models, such as caries management by risk assessment (CAMBRA), guides policymakers toward more efficient and patient-centered care.
- Formulation of regulatory frameworks and guidelines to evaluate the efficacy, equity, and ethical deployment of AI systems in dentistry. 47
